# Ultra‐widefield retinal imaging in Down syndrome: A high‐risk population for Alzheimer's disease

**DOI:** 10.1002/alz.093329

**Published:** 2025-01-09

**Authors:** Imre Lengyel, Jamie Mitchell, Adam Threlfall, Jessica Beresford‐Webb, Madeleine Walpert, Tony Holland, Tom MacGillivray, Tunde Peto, Lajos Csincsik

**Affiliations:** ^1^ Queen's University Belfast, Belfast United Kingdom; ^2^ University of Edinburgh, Edinburgh United Kingdom; ^3^ University of Cambridge, Cambridge United Kingdom; ^4^ Alzheimer's Research UK, Cambridge United Kingdom

## Abstract

**Background:**

People with Down syndrome (pwDS) have a high prevalence of early‐onset Alzheimer’s disease (AD). With the two‐fold increase in life expectancy for pwDS, identifying early biomarkers for AD in this patient population is needed (PMID: 32593336). Previously we have shown that phenotyping using ultra‐widefield retinal imaging (UWFI) has the potential to identify peripheral retinal biomarkers for AD, such as higher prevalence of peripheral hard drusen (pHD) and lower peripheral retinal vascular fractal dimension (RVFD) compared to controls (Ctrl) (PMID: 29621759, PMID: 34458553). In this cross‐sectional, case‐control study, the feasibility and the utility of UWFI in pwDS was investigated.

**Method:**

UWFI was performed on 24 pwDS and 17 cognitively healthy, age‐ and sex‐matched Ctrl. The images were graded for central and peripheral retinal vascular changes using the VAMPIRE software.

**Result:**

After quality control, 39 images of 20 patients and 34 images of 17 Ctrl were included in the study. pHD were detected on most of the images across both groups with no significant difference between pwDS and Ctrl. There was a higher arterial RVFD in the central zone (P<0.001) but no difference was observed in the peripheral zone. The venular RVFD were higher in the central zone (P<0.005) but lower in the peripheral zone (P<0.001) in pwDS compared to Ctrl. These results were the same with those diagnosed with and without AD. The arteriolar and venular width gradients were both significantly decreased in pwDS compared to Ctrl (P<0.02). This difference was only present in those with AD diagnosis.

**Conclusion:**

This study proved that UWFI is feasible in pwDS, and there are vascular changes that are associated with DS, especially in those with the diagnosis of AD. Therefore, we propose that peripheral retinal vasculature changes may hold a potential to monitor the progression of AD in this patient population.

